# High prevalence of pre-treatment and acquired HIV-1 drug resistance mutations among non-citizens living with HIV in Botswana

**DOI:** 10.3389/fmicb.2024.1338191

**Published:** 2024-02-20

**Authors:** Patrick T. Mokgethi, Wonderful T. Choga, Dorcas Maruapula, Natasha O. Moraka, Kaelo K. Seatla, Ontlametse T. Bareng, Doreen D. Ditshwanelo, Graceful Mulenga, Terence Mohammed, Pearl M. Kaumba, Moses Chihungwa, Tafireyi Marukutira, Sikhulile Moyo, Catherine K. Koofhethile, Diana Dickinson, Sununguko W. Mpoloka, Simani Gaseitsiwe

**Affiliations:** ^1^Botswana Harvard AIDS Institute Partnership, Gaborone, Botswana; ^2^Department of Biological Sciences, University of Botswana, Gaborone, Botswana; ^3^Center of Epidemic Response and Innovation, Faculty of Data Sciences, Stellenbosch University, Cape Town, South Africa; ^4^School of Allied Health Professionals, Faculty of Health Sciences, University of Botswana, Gaborone, Botswana; ^5^Independence Avenue Clinic, Gaborone, Botswana; ^6^Public Health, Burnet Institute, Melbourne, VIC, Australia; ^7^Department of Epidemiology, School of Public Health and Preventive Medicine, Monash University, Melbourne, VIC, Australia; ^8^Department of Immunology and Infectious Diseases, Harvard T.H. Chan School of Public Health, Boston, MA, United States; ^9^School of Health Systems and Public Health, University of Pretoria, Pretoria, South Africa; ^10^Division of Medical Virology, Faculty of Medicine and Health Sciences, Stellenbosch University, Tygerberg, South Africa

**Keywords:** HIV-1C, antiretroviral therapy (ART), drug resistance mutations (DRMs), non-citizens, Botswana, pre-treament drug resistance (PDR), acquired HIV drug resistance (ADR)

## Abstract

**Background:**

Approximately 30,000 non-citizens are living with HIV in Botswana, all of whom as of 2020 are eligible to receive free antiretroviral treatment (ART) within the country. We assessed the prevalence of HIV-1 mutational profiles [pre-treatment drug resistance (PDR) and acquired drug resistance (ADR)] among treatment-experienced (TE) and treatment-naïve (TN) non-citizens living with HIV in Botswana.

**Methods:**

A total of 152 non-citizens living with HIV were enrolled from a migrant HIV clinic at Independence Surgery, a private practice in Botswana from 2019–2021. Viral RNA isolated from plasma samples were genotyped for HIV drug resistance (HIVDR) using Sanger sequencing. Major known HIV drug resistance mutations (DRMs) in the *pol* region were determined using the Stanford HIV Drug Resistance Database. The proportions of HIV DRMs amongst TE and TN non-citizens were estimated with 95% confidence intervals (95% CI) and compared between the two groups.

**Results:**

A total of 60/152 (39.5%) participants had a detectable viral load (VL) >40 copies/mL and these were included in the subsequent analyses. The median age at enrollment was 43 years (Q1, Q3: 38–48). Among individuals with VL > 40 copies/mL, 60% (36/60) were treatment-experienced with 53% (19/36) of them on Atripla. Genotyping had a 62% (37/60) success rate – 24 were TE, and 13 were TN. A total of 29 participants (78.4, 95% CI: 0.12–0.35) had major HIV DRMs, including at least one non-nucleoside reverse transcriptase inhibitor (NNRTI) associated DRM. In TE individuals, ADR to any antiretroviral drug was 83.3% (20/24), while for PDR was 69.2% (9/13). The most frequent DRMs were nucleoside reverse transcriptase inhibitors (NRTIs) M184V (62.1%, 18/29), NNRTIs V106M (41.4%, 12/29), and K103N (34.4%, 10/29). No integrase strand transfer inhibitor-associated DRMs were reported.

**Conclusion:**

We report high rates of PDR and ADR in ART-experienced and ART-naïve non-citizens, respectively, in Botswana. Given the uncertainty of time of HIV acquisition and treatment adherence levels in this population, routine HIV-1C VL monitoring coupled with HIVDR genotyping is crucial for long-term ART success.

## 1 Introduction

Notwithstanding the enormous achievements reported since the introduction of antiretroviral therapy (ART), unequal and limited access to HIV treatment services and prevention interventions for marginalized and migratory populations hamper the global HIV response to end AIDS as a public health threat by 2030 (Schultz, 2014; Tanser et al., 2015; IN DANGER: UNAIDS Global AIDS Update, 2022; The path that ends AIDS: UNAIDS Global AIDS Update, 2023). The emergence of HIV drug resistance (HIVDR) also poses a challenge to the success of ART (Rowley et al., 2016; Bertagnolio et al., 2017; Shafer and Frenkel, 2019; HIV DRUG RESISTANCE REPORT, 2021; The path that ends AIDS: UNAIDS Global AIDS Update, 2023). The burden of the global HIV epidemic is concentrated in Eastern and Southern Africa, where 20.4 million of 39 million people living with HIV (PLWH) reside (The path that ends AIDS: UNAIDS Global AIDS Update, 2023). Botswana bears the brunt of the HIV epidemic with the third highest HIV prevalence standing at 20.8% of people aged 15–64 years living with HIV (Government of Botswana, 2023). Botswana, an upper middle-income country, hosts a substantial population of immigrants from neighboring HIV-epidemic countries, including South Africa, Zimbabwe, and Zambia (Marukutira et al., 2018, 2019a,b; Fennell et al., 2023). Currently, Botswana is home to ~110,000 non-citizens, of whom an estimated 30,000 are living with HIV, yielding a prevalence of 27.27% (95% CI: 24.32–30.77%) (Marukutira et al., 2018, 2019a; McAuliffe et al., 2021). The majority of these individuals migrate from neighboring countries with high HIV risk, such as Zimbabwe, Zambia, and South Africa (with 12.9, 11.1, and 19.1%) HIV prevalence, respectively. In 2020, the non-citizen population in Botswana accounted for 110,268 inhabitants. 43% of them were female, whereas 57% were male. The most represented nationalities among them were Zimbabwean (58.31%), followed by South African (5.20%), Indian (5.12%), Chinese (4.33%), and Zambian (4%) (Summary Sheet, 2020; Migrant-refugees, 2023). This is considered an underestimate given the influx of putatively undocumented non-citizens entering Botswana and could potentially impede efforts in HIV prevention and control (Marukutira et al., 2020).

Migration has contributed to increasing health challenges amongst non-citizens, including vulnerability to HIV acquisition and other sexual health issues globally (Lurie and Williams, 2014; Marukutira et al., 2018; Kate Grabowski et al., 2020; McAuliffe et al., 2021). It has long been established that population mobility plays a critical role in the dynamics of HIV/AIDS throughout Sub-Saharan Africa (SSA) and findings show that non-citizens are indeed more at risk of HIV infection than natives (Anglewicz, 2012; Schultz, 2014; Song et al., 2023). Understanding HIV molecular epidemiology and drug-resistance mutations in this vulnerable population is crucial due to their suboptimal HIV treatment and care. Botswana has exceeded and aims to maintain the UNAIDS 95-95-95 targets to end the AIDS epidemic by 2030 (Mine et al., 2021). However, despite the country’s remarkable results from the HIV program at the national level, some gaps remain, such as limited access to ART and HIV care to non-citizens (Gaolathe et al., 2016; Marukutira et al., 2018; Mine et al., 2021).

Monitoring of HIVDR boosts the long-term effectiveness of ART (Yebra et al., 2013; Wymant et al., 2022; Zhao et al., 2022). Global expansion of access to ART and monitoring optimized outcomes of PLWH, minimize transmission of drug-resistant HIV and ensure the sustainability of ART programs (Marukutira et al., 2020; Wymant et al., 2022; Zhao et al., 2022). Furthermore, genotypic resistance testing (GRT) offers insight into the HIV molecular epidemiology within the population (de Felipe et al., 2011; Maggiorella et al., 2020). The information obtained can guide public health decisions to enhance treatment, care, and coverage, ultimately aiding Botswana in achieving substantial viral suppression and reducing HIV incidence throughout the population (de Felipe et al., 2011; Yebra et al., 2013; Andersson et al., 2018; Maggiorella et al., 2020; Wymant et al., 2022). In this study, we aimed to characterize HIV drug resistance mutations (DRMs) profiles among non-citizens living with HIV in Botswana.

## 2 Methods and materials

### 2.1 Study design and population

This was a retrospective cross-sectional study utilizing 152 residual samples collected between 2019 and 2021 from non-citizens living with HIV-1 in Botswana. We defined the non-citizen population as people born outside their country of residence. Eligible participants were adults older than 18 years seeking medical care at the Independence Surgery, a private clinic in Gaborone, Botswana. The Independence Surgery clinic specializes in providing medical services to a large number of non-citizens living with HIV in Botswana.

**Figure 1:**
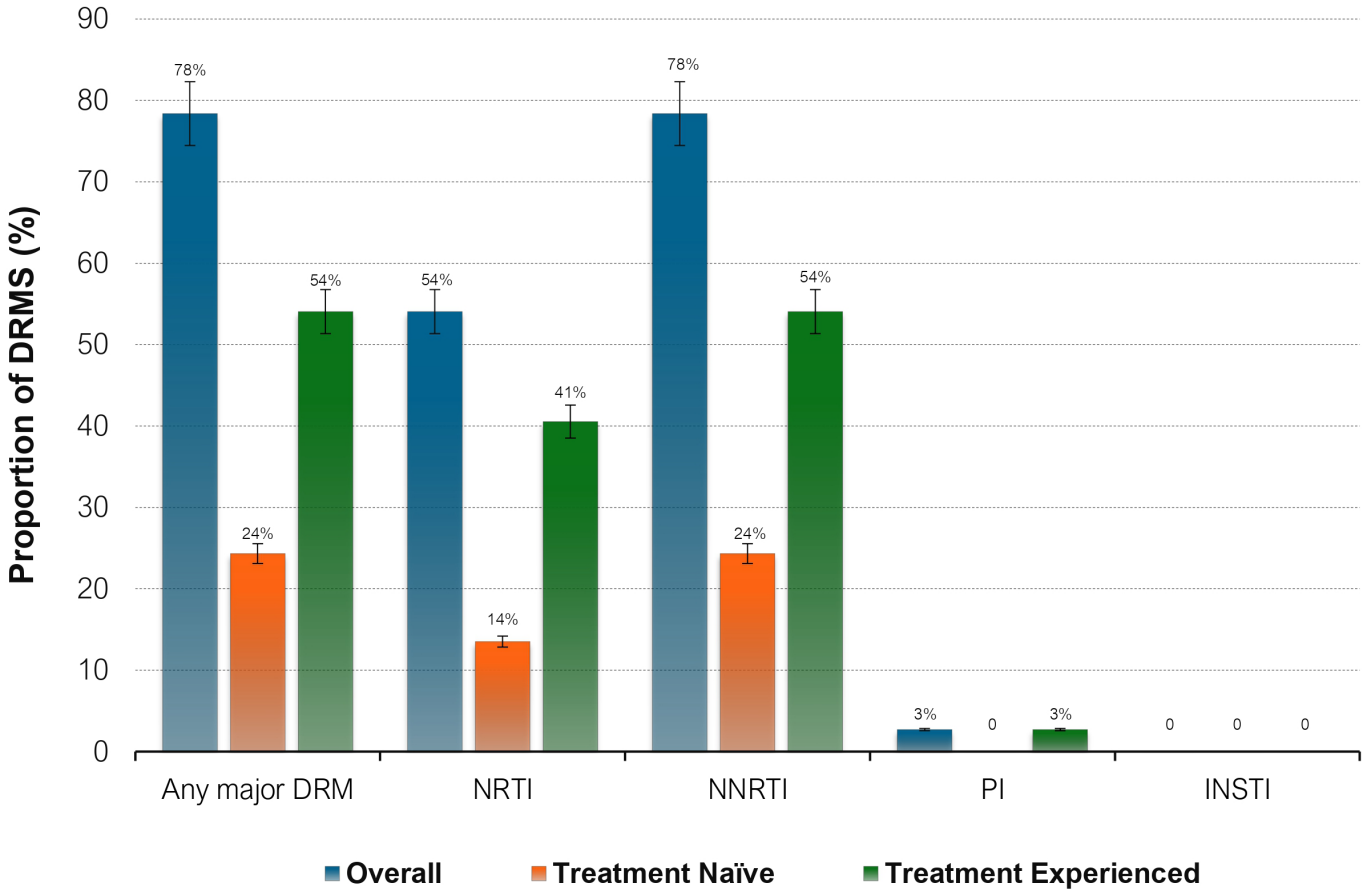
Proportions of HIV drug resistance mutations by ART status. NNRTI, non-nucleoside reverse transcriptase inhibitors; NRTI, nucleoside reverse transcriptase inhibitors; PI, protease inhibitors; INSTI, integrase strand transfer inhibitors; DRM, drug resistance mutations.

Participants were included if they had at least one visit of HIV viral load (VL) testing and their socio-demographic (age, sex, country of origin, and attending hospital) data were compiled. A total of 60 (39.5%) out of 152 samples had detectable viral loads (VL >40 copies/mL) and were used for the current study (See Figure 1).

### 2.2 Viral load quantification

**Figure 2:**
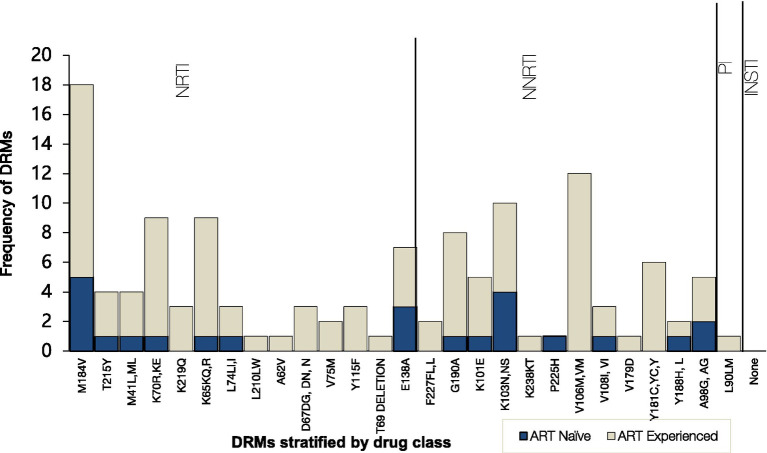
Frequency of HIV Drug resistance mutations to the main antiretroviral classes stratified by ART status. NNRTI, nonnucleoside reverse transcriptase inhibitors; NRTI, nucleoside reverse transcriptase inhibitors; PI, protease inhibitors; INSTI, integrase strand transfer inhibitors.

HIV viral load testing from plasma specimens was performed using the Abbott m2000sp/Abbott m2000rt platform (Wiesbaden, Germany), Cobas TaqMan/Cobas Ampliprep HIV-test (Roche Molecular Systems, Branchburg, NJ, United States) at Botswana Harvard Health Partnership (BHP) according to the manufacturer’s instructions. At the time of enrolment VF was defined as two consecutive VLs greater than 400 copies/mL and virologic suppression as a viral load <400 copies/mL as per national ART guidelines. The lower limit of detection (LOD) in plasma is 40 copies/mL for this VL detection platform (See Figure 2).

### 2.3 Genotypic resistance testing

#### 2.3.1 Nucleic acid extraction

HIV-1 total nucleic acid (RNA/DNA) was extracted from 300 μL of plasma samples with VL > 40 copies/mL using the BioMérieux Nuclisens easy-mag platform according to manufacturer’s instructions (Biomérieux, Marcy I’Etoile, France). The isolated RNA was used as a first-round PCR template and stored at −80°C for future use.

#### 2.3.2 Amplification of HIV-1 *protease* and *reverse transcriptase* regions

Using in-house genotypic resistance testing (Seatla et al., 2019; Bareng et al., 2022), the extracted RNA was amplified by nested Polymerase chain reaction (PCR) to obtain the HIV-1 *pol* which comprised the entire protease (PR) gene (codons 1-99) and reverse transcriptase (RT) gene (codons 1-560). The RT-PCR was done using the following primers (Rowley et al., 2016; Bareng et al., 2022).

Both one-step RT-PCR and nested PCR amplifications were carried out in the Applied Biosystems 2,720 Thermal Cycler. The RT-PCR was performed using Roche one-step transcriptor enzyme while the nested PCR was done using Phusion High Fidelity enzyme (Rowley et al., 2016; Bareng et al., 2022; Kelentse et al., 2023).

#### 2.3.3 Amplification of HIV *integrase* region

The HIV integrase (IN) gene (codons 1-288) was amplified using nested PCR with an RT step to generate complementary DNA (cDNA). Both one-step RT-PCR and nested PCR amplifications were carried out using Applied Biosystems 2,720 Thermal Cycler. Primers used for the first and second rounds were adopted from the inhouse protocol, (Seatla et al., 2019; Bareng et al., 2022) without modification.

#### 2.3.4 Confirmation of amplification

Amplification was confirmed by electrophoresis in a 1% agarose gel prepared using Tris-Borate-EDTA (TBE) buffer. The gel was stained with 5 μL ethidium bromide (0.5 mg/mL) and ran at 90 volts for 45 min. Generated amplicons were visualized under a UV source (260 nm). Successfully amplified PCR amplicons were purified using Applied Biosystems ExoSAP-IT PCR product cleanup reagent (Waltham, Massachusetts, United States) according to the manufacturer’s instructions (Bareng et al., 2022).

#### 2.3.5 Sequencing

Sequences were generated using BigDye sequencing chemistry on an ABI 3130xl Genetic Analyzer (Applied Biosystems, Foster City, Canada) using previously described cycle sequencing primers covering for both HIV *protease* and *reverse transcriptase* regions (Rowley et al., 2016). The following sequencing primers were used for HIV *protease*, *reverse transcriptase* and *integrase* region:

The cycle sequencing reaction mix constituted 4.8 μL of RNase-free water, 3 μL of Big Dye 5X sequencing buffer, 1 μL BigDye terminator, 0.2 μL of 2 μM of each sequencing primer, and 1 μL of the purified amplicon, to make a total reaction volume of 10 μL. The cycle sequencing reaction conditions were as follows: 25 cycles at 96°C for 10 s, 50°C for 5 s, 60°C for 4 min and hold at 4°C. The BigDye XTerminator purification kit (Applied Biosystems, Foster City, United States) was used to purify the sequencing reaction by adding 10 μL of the BigDye XTerminator and 45 μL SAM solution to cycle sequencing products. The reaction plate was vortexed at 1800 rpm for 45 min and then centrifuged at 3000 g for 3 min at room temperature before loading the plate in the genetic analyzer (Rowley et al., 2016; Seatla et al., 2019; Bareng et al., 2022).

#### 2.3.6 Sequence and mutation analysis

Electropherograms were manually edited using Sequencher v5.0 software (Gene Codes Corp., Ann Arbor, MI, United States). Where necessary, the low-quality ends of chromatograms were trimmed before assembly. The subsequent fasta files were further assessed using Aliview version. 1.26 prior to mutation analysis. We used a muscle algorithm implemented in Aliview ver. 1.26 and the HXB2 pol nucleotide sequence to perform the full alignment. The GeneCutter tool implemented in the HIV Los Alamos HIV database^1^ was used to extract PR and RT regions from the alignment. PR and RT nucleotide sequences were aligned using the ClustalW algorithm implemented in Bioedit to characterize HIV-1 variants. Viral sequences included the HIV *pol* gene (protease, codons 1-99, reverse transcriptase, codons 1-247, and integrase, codons 1-288). The generated consensus sequences were loaded into the Stanford HIV database^2^ for the determination of HIV drug-resistance mutations. IQ-Tree version 1.6.12-Linux was used to generate a phylogenetic tree which was visualized in FigTree v1.4.4. The 37 sequences generated from this study are available in GenBank under accession numbers OR548006–OR548042.

### 2.4 Statistical analysis

Statistical analyses were conducted using Stata SE 15 (Stata Corp, College Station, TX, United States). Descriptive statistics were used to summarize variables. Categorical variables (gender and country) were reported as percentages and continuous (VL and age) as medians with interquartile ranges. The statistical significance was calculated using the Fisher exact test or Chi-square for categorical variables and the Mann–Whitney test for continuous variables. The proportions of HIV DRM were estimated using binomial exact methods and compared with the comparison of proportion tests among two ART groups. A confidence level of 95% was chosen with *p*-values <0.05 considered statistically significant.

### 2.5 Ethics statement

The local Ethics Committee approved this research, the Health Research and Development Division (HRDC) (Reference number HPRD: 6/14/1) of the Botswana Ministry of Health and the University Botswana Institutional Review Board (IRB). Being a retrospective study done from residual samples, the ethics committee has authorized its conduct without the need to obtain a specific informed consent from the participants. Data was processed using unique identifiers to ensure confidentiality (See Tables 1–3).

**Table 1:** RT-PCR primers.

PCR round	Primer name	Primer sequences
First	CWF1-LNA2 (forward) (2 μM)	5′-GAA GGA CCA AAT GAA AGA YTG-3’
	CWR1-LNA3 (reverse) (2 μM)	5’-GCATAC TTY CCG TTT TCAG-3’
Second (nested)	CWF1-LNA2 (forward) (2 μM)	5′-GAA GGA CCA AAT GAA AGA YTG-3’
	RT-20C (reverse) (2 μM)	5’CTG CCA ATT TCT AAC TGC CTT C-3’

**Table 2:** Integrase PCR primers.

PCR round	Primer name	Primers
First	INFORI (forward)	5′ GGA ATC ATT CAA GCA CAA CCA GA 3′
	INREV-1 (reverse)	5’-TCT CCT GTA TGC AGA CCC CAA TAT-3’
Second	HIV-4141 (forward)	5′ GGA ATC ATT CAA GCA CAA CCA GA 3′
	INREV-II (reverse)	5’ CCT AGT GGG ATG TGT ACT TCT GA 3′

**Table 3:** Sequencing primers.

HIV gene	Primer name	Primers
PR-RT	CWF1	5^′^-GAAGGACACCAAATGAAAGAYTG-3′
	CWCS2	5^′^ -AGAACTCAAGA CTTTTGGG-3^′^
	CWCS3	5^′^ -TGCTGGGTGCGGTATTC-3^′^
	CWCS5	5^′^ -TGGTAAATTTGATATGTCCAT-3^′^
	Seq2.1-F2	5^′^-GGCCAGGGAATTTTCTTCAGAGC-3^′^
	Seq6	5^′^-CCATCCCTGTGGAAGCACATTA-3^′^
	RT-20C	5′ -CTGCCAATTCTAATTCTG CTTC-3′
Integrase	HIV + 4,141	5’-TCT ACC TGG CAT GGG TAC CA-3’
	INREV-1	5’-TCT CCT GTA TGC AGA CCC CAA TAT-3’
	INFORI	5′-GGA ATC ATT CAA GCA CAA CCAGA-3
	INREVII	5′-CCT AGT GGG ATG TGT ACT TCT GA-3’
	IN4764AS	5’C CAT GTA CTG CTG TCT TAB-3’

## 3 Results

### 3.1 Participants characteristics

The overall median age at enrollment of the study participants was 43 years [interquartile range (Q1, Q3: 38, 48)] and the majority of the study participants were women (97/152, 64%), summarized in Table 4. Among the 152 participants, 149 (98%) had known ART status (either treatment experienced, TE or treatment-naïve, TN). The majority of the participants, 118 (78%) were on treatment, 31 (20%) were treatment-naïve at the time of sampling whereas 3 (2%) were of unknown ART status. Among the TE 118 participants, the ART regimen was known for 86 (73%) participants. Of all 152 participants, 147 (97%) had HIV-1 viral load data and 94(64%) were virally suppressed (HIV-1 VL ≤ 400 copies/mL). The median HIV-1 VL among TN individuals (3.8 [Q_1_, Q_3_: 2.2, 4.7] log_10_ copies/mL) which was less than virally unsuppressed participants (4.4 [Q_1_, Q_3_: 3.7, 4.8] log_10_ copies/mL).

**Table 4:** Overall characteristics of study participants.

Characteristic	Total (*n* = 152)	Viral Suppression (<400 copies/mL) on ART (*n* = 94)	Viral unsuppressed (>400 copies/mL) on ART (*n* = 27)	Treatment-naïve *n* = 31	*p* value
Gender, *n* (%)					
Female	97 (63.8)	63 (67.0)	14 (51.9)	20 (64.5)	0.4
Male	53 (34.9)	29 (30.9)	13 (48.1)	11 (35.5)	
unknown	2(1.3)	2 (2.1)	0	0	
Age [*n* (%)]					
<30	16 (10.5)	7 (7.4)	2(7.5)	7 (22.6)	0.1
30–45	77 (50.7)	48 (51.1)	12(44.4)	17 (54.8)	
>45	56(36.8)	37(39.4)	12 (44.4)	7 (22.6)	
Unknown	3(2.0)	2(2.1)	1(3.7)	0	
Treatment type (*n* = 121), *n* (%)					
Atripla (EFV/TDF/FTC)	62 (51.2)	47 (50.0)	15 (55.6)		<0.01
Unknown	35 (28.9)	28 (29.8)	7 (25.9)	n/a	
TLD (DTG/3TC/TDF)	19 (15.8)	15(16.0)	4(14.8)		
Dovato (DTG/3TC)	4 (3.3)	3 (3.2)	1 (3.7)		
Truvada (FTC/TDF)	1 (0.8)	1 (1.0)	0		
Country of origin *n* (%)					
Zimbabwe	142 (93.4)	88 (93.6)	25 (92.6)	29 (93.5)	0.5
Other	8 (5.3)	4 (4.3)	2 (7.4)	2 (6.5)	
Unknown	2 (1.3)	2(2.1)			
HIV-1 RNA (log_10_ Copies/mL), Median (Q1, Q3)	1.6 (1.6–3.6)	1.6 (1.6–1.6)	4.4 (3.7–4.8)	3.8 (2.2–4.7)	<0.01

Demographical, clinical, and virological characteristics of the study group, stratified by ART status and level of suppression. Continuous variables values are expressed as median and first and third quartiles (Q1, Q3) (25–75 percentiles) and categorical variables are displayed as the number of cases (percentage and confidence interval-95% in parentheses). VL (viral load in Log10). Other (Malawi, South Africa, Tanzania, Mozambique, and Zambia).

### 3.2 Characteristics of non-citizens by ART status with detectable viremia

A total of 60/152 (39.5%) participants had a detectable viral load (VL) >40 copies/mL and these were included in the subsequent analyses. The baseline characteristics of the 60 non-citizen participants with detectable viral load are summarized in Table 5. Of the 60 participants living with HIV with a detectable VL (>40 copies/mL), 45 (75%) of them had VL >400 copies/mL whereas 15 (25%) samples had VL <400copies/mL. Of all 60 participants, 36 participants were TE 23 were TN, and one participant had an unknown status. Most of TE participants were on Atripla 53% (19/36). The majority, (58%) of the participants were female.

**Table 5:** Characteristics of non-citizens by ART status with detectable viremia.

	Overall *N* = 60	TE *N* = 36	TN *N* = 23	*p* value
Age, Median (Q1, Q3)	43 (37, 47)	43 (39, 47)	39 (30, 45)	0.02
Gender				
Male, *N* (%)	24 (40)	16 (67)	8(33)	0.96
Female, *N* (%) Unknown	35 (58) 1 (2)	20(57)	15 (43)	
Geographical Origin				
Mozambique, *N* (%)	1 (0.7)	0	1	
Tanzania, *N* (%)	2 (1.3)	2	0	
Zimbabwe, *N* (%)	57(95)	34(60)	22(40)	
HIV-1 RNA (Log10 Copies/mL), Median (Q1, Q3)	3.7 (1.7, 5.9)	4.0(2.6, 4.7)	3.9(2.8, 4.8)	0.66

Demographical, clinical, and virological characteristics of the study group with detectable viremia, stratified by ART status. Continuous variables values are expressed as median and first and third quartiles (Q1, Q3) (25–75 percentiles) and categorical variables are displayed as the number of cases (percentage and confidence interval-95% in parentheses). VL (viral load in Log10), TE (Treatment experienced), TN (Treatment Naive), N. Detectable viremia was defined as an HIV RNA level ≥ 40 copies/mL.

The median age at enrollment was 43 years (Q_1_, Q_3_: 37–47), and there was a significant difference in median age for TE participants [43 years (Q_1_, Q_3_: 39–47)] compared to TN participants [39 years (Q_1_, Q_3_: 30–45)] (*p* = 0.02). The overall median log10 HIV-1 RNA viral load was 3.7 (Q_1_, Q_3_: 1.7–5.9). The median log10 HIV-1 RNA viral load for TE was 4.0(Q_1_, Q_3_: 2.6, 4.7) whilst the median log10 HIV-1 RNA viral load for TN was 3.9 (Q_1_, Q_3_: 2.8, 4.8). Regarding treatment status, TE was not statistically significant to participants who had not started ART (*p* = 0.66).

### 3.3 HIV genotyping and associated drug-resistant mutations

We successfully sequenced 37 out of 60 (62%) samples; 24 were TE and 13 were TN. Of all the 37 successfully sequenced samples had HIV-1 RNA viral load >400 copies/mL. 23 out of the 60 (38%) samples were unsuccessful for sequencing, 11 were TN and 12 were TE. Among these 23 participants, 9 (39%) had HIV-1 RNA viral load >400 copies/mL (5 were TN and 4 were TE) whereas 14 (61%) had an HIV RNA viral load below 400 copies/mL, (5 were TN and 9 were TE).

All sequences were HIV-1 subtype C (HIV-1C) and the amplified fragment covered the HIV-1 Pol region, partial RT and PR. 29 out of 37, (78%) of the participants were reported to have any major mutation among them, 54% (20/37) of participants also had NRTI DRMs, sequences with any NNRTI mutations were found in 78% of participants (*n* = 29). Mutations both to NRTIs and NNRTIs were detected in 54% of the participants (*n* = 20). Only one participant had PI DRM and no major INSTI-associated DRMs were observed (See Table 6).

**Table 6:** Overall prevalence of HIV-1 drug resistance by drug group.

DRMs	*N* out of 37	Percentage, %
NRTI	20	54
NNRTI	29	78
PI	1	3
INSTI	0	0
NRTI and NNRTI	20	54
NRTI, NNRTI, and PI	1	3

NNRTI, non-nucleoside reverse transcriptase inhibitors; NRTI, nucleoside reverse transcriptase inhibitors; PI, protease inhibitors; INSTI, integrase strand transfer inhibitors; DRMs, drug resistance mutations.

### 3.4 Proportions of HIV drug resistance mutations by ART status

When stratified by ART status, a higher prevalence of major DRMs was observed in TE 20/24 (83%) than in TN 9/13 (69%) (*p* = 0.32). Amongst the TN group, overall DRM prevalence was 69% (95% CI 38.5–90.9) of which 38.5% had major DRMs to NRTIs and 69% to NNRTIs. The overall most prevalent DRMs were NRTI M184V (18/29, 62%), followed by NNRTIs V106M (12/29, 41%) and K103N (10/29, 34%). NRTI mutation M184V mutation was most prevalent in both TN (5/9, 56%) and TE (13/20, 65%) whilst NNRTI mutation K103N was most prevalent in both TN (4/9, 44%) and TE (6/20, 30%). V106M, one of the most prevalent mutations, was observed only in TE participants (12/20, 60%). Only one participant had PI DRM: L90M, and none of the participants had INSTI-associated resistance mutations.

## 4 Discussion

This is the first study to assess the HIV molecular landscape of non-citizens living with HIV in Botswana. Previous studies were conducted in a few areas providing only a partial picture of the virologic outcomes in this population (Marukutira et al., 2017, 2018, 2019b; McAuliffe et al., 2021). The present retrospective cross-sectional study describes the prevalence of acquired and pre-treatment HIVDR mutations among treatment-experienced and treatment-naïve non-citizens living with HIV in Botswana. We report a relatively high prevalence of HIV drug resistance among non-citizens, with a higher prevalence of resistance to NNRTI, NRTI and a low prevalence of resistance to PI and none to INSTI-based regimens. Similarly, non-citizens who are TE harbored higher HIV drug resistance than TN individuals, however, the difference was not statistically significant.

We report high HIV-1 ADR and PDR prevalence among non-citizens living with HIV-1 in Botswana. However, according to some studies done in Botswana, (Moyo et al., 2019), a low prevalence of PDR was reported where the prevalence of NRTI-associated or NNRTI-associated DRMs among these individuals was 15.9 and 32.6%, respectively (Moyo et al., 2019; Maruapula et al., 2022). The majority of the study participants were from Zimbabwe (Migrant-refugees, 2023). Zimbabwe is one of the countries in Southern Africa most affected by the HIV epidemic with HIV prevalence of 12.9% (Chowdhury et al., n.d.). However, Zimbabwe has made significant strides in scaling up access to HIV testing and treatment Drug resistance is a serious threat to the global scale-up of HIV treatment particularly in many resource-limited settings like Zimbabwe with limited ART treatment options. Zimbabwe has also adopted the use of dolutegravir (DTG) in combination with tenofovir and lamivudine (TLD). A prospective cohort study was conducted between October 2021 and April 2023 among antiretroviral therapy (ART) naïve adults (≥18 years) attending care at an HIV clinic in Zimbabwe. DRM prevalence of 19% was observed and was found to be more prevalent among prior treatment-exposed participants compared to treatment-naïve participants (27% vs. 17%) (Vinie Kouamou, 2021; Kouamou et al., 2023).

The high prevalence of HIV drug-resistance mutations among non-citizens found in our study is in concordance with previous similar studies that demonstrated migration to be a factor for increased risk of HIV acquisition, poor treatment and adherence leading to poor virologic outcomes in non-citizens living with HIV (Novitsky et al., 2013; Gaolathe et al., 2016; Marukutira et al., 2017, 2019a; Shafer and Frenkel, 2019; Mine et al., 2021). Treatment-experienced participants may not have been consistently adhering to their prescribed medication regimen and the effectiveness of the treatment may have been compromised. Moreover, as it is the case for most non-citizens, treatment-experienced participants who were not virally suppressed due to poor adherence may have presented to care at a later stage. Delayed resistance testing and proper HIV care and treatment can result in higher viral loads and poor adherence may allow the virus to replicate thereby increasing the viral load. It also could not be ruled out that some of the participants reporting to be treatment naïve were actually on treatment as has been documented elsewhere (Marukutira et al., 2017; Faturiyele et al., 2018; Marukutira et al., 2019b; Maggiorella et al., 2020). In agreement with our observations, a study in Portugal that aimed to identify long-term trends in HIV-1 molecular epidemiology and antiretroviral drug resistance among 5,177 non-citizens followed between 2001 and 2017 found the prevalence of HIV DRM among non-citizens to be high and increased over time (Shafer and Frenkel, 2019; Pimentel et al., 2020; Ji, 2022). Another study in Sweden aimed to assess the trends of transmitted drug resistance (TDR) in HIV-1 from 1713 participants newly diagnosed from 2010–2016 where 1,225 were non-citizens, and 522 of the 1,225 participants were from sub-Saharan Africa. They observed a significant increase in NNRTI TDR from 1.5% in 2010 to 6.2% in 2016 in the non-citizen population, indicating the need to continue monitoring HIV DRM profiles in non-citizens (Andersson et al., 2018; Shafer and Frenkel, 2019). These studies further concluded that HIV molecular epidemiology in migrants suggested high levels of connectivity with their country of origin and lifestyle post-migration (Shafer and Frenkel, 2019; Pimentel et al., 2020; Ji, 2022).

Overall, NRTI mutation M184V was the most prevalent (62%) followed by NNRTI mutations V106M (41%) and K103N (34%). The main drug resistance mutations affecting the development of HIV-1 resistance to NRTIs and NNRTIs occurred at a high rate. This is not surprising since almost half (41%) of the participants had post-exposure to Atripla, which is probably the reason for the highest levels of resistance to NRTIs (emtricitabine and lamivudine) (54%, *n* = 20) and NNRTIs (nevirapine and efavirenz) (78%, *n* = 29). Only 16% of non-citizens were on a Dolutegravir-containing regimen, while 43% of the participants’ regimen was unknown. The findings of this study were high compared to the low prevalence of NRTI-associated (16%) and NNRTI-associated (33%) resistance mutations among residents of rural and peri-urban communities across Botswana (Moyo et al., 2019). Only 78% of the non-citizens on ART were found to control viremia which is significantly lower than the 98% of the Botswana native individuals living with HIV and controlling viremia (Mine et al., 2021). The difference could be explained by a greater difficulty of non-citizens to access health care and consequently, by lower compliance to therapy (Marukutira et al., 2017, 2018, 2019b). Moreover, this is in line with data confirming an elevated risk of virologic failure to the antiretroviral regimen in non-citizens living with HIV as compared to the citizens. Due to the aforementioned challenges, non-citizens are likely to have higher HIV DRMs (Gonzalez-Serna et al., 2014; Andersson et al., 2018; Marukutira et al., 2019b). Additionally, delayed initiation to HIV treatment and care could contribute to poor virological outcomes, accumulation and transmission of HIV drug resistance mutations among non-citizens, which would exacerbate the local HIV epidemic (de Felipe et al., 2011; Pimentel et al., 2020; Wymant et al., 2022).

Our results provide the most recent data regarding the HIV-1 drug resistance profile in non-citizens living with HIV in Botswana, compared to previous similar studies on non-citizens which were conducted only on the clinical outcomes of a disproportionate HIV treatment and care of non-citizens living with HIV in Botswana (Marukutira et al., 2018; Escudero et al., 2019; Marukutira et al., 2019b). This study had limitations which included missing information such as previous ART regimens, date of ART switch, lack of adherence data, duration of their stay in the country, whether the infection was pre-or post-migration, time of HIV diagnosis and duration on ART which limited the interpretations of their presentations towards HIV care, transmission and acquisition of HIV drug resistance. The small sample size of treatment-naïve participants and low amplification rate may have been a limiting factor concerning our findings. 23 out of the 60 (38%) samples were unsuccessful for sequencing. Among these 23, 14 (61%) had HIV-1 RNA viral load <400 copies/mL. Guidelines from most clinical laboratories advise that HIV drug resistance testing be performed on specimens with viral loads of at least 1,000 copies of HIV RNA/mL. This practice was based on the rationale that successful sequencing diminished in efficiency with diminishing levels of RNA. The lower HIV plasma viral load could potentially have hindered the amplification process, thereby contributing to the lower sequencing success rate (Cane et al., 2008; Richman, 2014). These highlight the use of highly sensitive genotyping assays for amplification of samples with viral load below 1,000 copies/mL. The use of proviral DNA plays a critical role when plasma has failed to amplify and high concordance in HIV drug resistance mutations in both plasma and proviral DNA have been reported. The study had 95% non-citizens from Zimbabwe, therefore it did not provide a great representativeness of non-citizens from different countries residing in Botswana. A larger cohort with all regions well represented is necessary to confirm our findings. However, our study provided important information on the molecular epidemiology landscape and resistance profile of the HIV-1 variants currently circulating among non-citizens in Botswana.

The main findings from our study are high HIV-1 ADR and PDR prevalence among non-citizens living with HIV in Botswana. Our study reported a 78% prevalence of any major HIV DRMs among the non-citizen population in Botswana among ART naïve and experienced individuals. This study highlights the importance of continuous HIV-1 drug resistance profiling among non-citizens living with HIV in Botswana coupled with specific public health interventions targeting the non-citizen population to alleviate poor virologic outcomes in both non-citizen and citizen populations (Andersson et al., 2018; Marukutira et al., 2019b; Wymant et al., 2022). This will contribute positively towards the country’s efforts to prevent further development and transmission of HIV drug-resistant variants, an intervention ideal for sustaining UNAIDS 95-95-95% targets and towards ending HIV by 2030 (Stover et al., 2021; Wise, 2023). The data we provide here is essential to guide HIV prevention and intervention strategies further extended to non-citizens (Grabowski et al., 2014; Grabowski and Redd, 2014; Gaolathe et al., 2016).

## Data availability statement

The datasets presented in this study can be found in online repositories. The names of the repository/repositories and accession number(s) can be found at: https://www.ncbi.nlm.nih.gov/genbank/, OR548006–OR548042.

## Ethics statement

The studies involving humans were approved by the local Ethics Committee, the Health Research and Development Division (HRDC) (Reference number HPRD: 6/14/1) of the Botswana Ministry of Health and the University Botswana Institutional Review Board (IRB). The studies were conducted in accordance with the local legislation and institutional requirements. The ethics committee/institutional review board waived the requirement of written informed consent for participation from the participants or the participants’ legal guardians/next of kin because this was a retrospective study.

## Author contributions

PM: Conceptualization, Data curation, Formal analysis, Investigation, Methodology, Project administration, Visualization, Writing – original draft, Writing – review & editing. WC: Conceptualization, Data curation, Formal analysis, Methodology, Resources, Software, Supervision, Validation, Visualization, Writing – review & editing. DM: Conceptualization, Supervision, Writing – review & editing. NM: Formal analysis, Methodology, Writing – review & editing. KS: Writing – review & editing. OB: Formal analysis, Methodology, Writing – review & editing. DoD: Writing – review & editing. GM: Data curation, Formal analysis, Writing – review & editing. TMo: Project administration, Resources, Writing – review & editing. PK: Writing – review & editing. MC: Project administration, Resources, Writing – review & editing. TMa: Conceptualization, Writing – review & editing. SMo: Conceptualization, Formal analysis, Funding acquisition, Investigation, Methodology, Resources, Supervision, Validation, Writing – review & editing. CK: Conceptualization, Supervision, Visualization, Writing – review & editing. DiD: Conceptualization, Investigation, Resources, Visualization, Writing – review & editing. SMp: Conceptualization, Methodology, Resources, Supervision, Writing – review & editing. SG: Conceptualization, Data curation, Formal analysis, Funding acquisition, Investigation, Methodology, Resources, Supervision, Validation, Visualization, Writing – review & editing.

## Footnotes

^1^ https://www.hiv.lanl.gov/content/sequence/GENE_CUTTER/cutter.html

^2^ http://hivdb.Stanford.edu/

## References

[ref1] Andersson E. Nordquist A. Esbjörnsson J. Flamholc L. Gisslén M. Hejdeman B.. (2018). Increase in transmitted drug resistance in migrants from sub-Saharan Africa diagnosed with HIV-1 in Sweden. AIDS 32, 877–884. doi: 10.1097/QAD.0000000000001763, PMID: 29369826

[ref2] Anglewicz P. (2012). Migration, marital change, and HIV infection in Malawi. Demography 49, 239–265. doi: 10.1007/s13524-011-0072-x, PMID: 22109083 PMC3787875

[ref3] Bareng O. T. Choga W. T. Maphorisa S. T. Seselamarumo S. Seatla K. K. Mokgethi P. T.. (2022). HIV-1C in-house RNA-based genotyping assay for detection of drug resistance mutations in samples with low-level viral loads. Inf. Drug Resistance 15, 7565–7576. doi: 10.2147/IDR.S388816, PMID: 36582452 PMC9792565

[ref4] Bareng O. T. Seselamarumo S. Seatla K. K. Choga W. T. Bakae B. Maruapula D.. (2022). Doravirine-associated resistance mutations in antiretroviral therapy naive and experienced adults with HIV-1 subtype C infection in Botswana. J Glob Antimicrob Resist 31, 128–134. doi: 10.1016/j.jgar.2022.08.008, PMID: 35973671 PMC9750894

[ref5] Bertagnolio S. Beanland R. L. Jordan M. R. Doherty M. Hirnschall G. (2017). The World Health Organization's response to emerging human immunodeficiency virus drug resistance and a call for global action. J. Infect. Dis. 216, S801–S804. doi: 10.1093/infdis/jix402 29040686 PMC5853942

[ref6] Cane P. A. Kaye S. Smit E. Tilston P. Kirk S. Shepherd J.. (2008). Genotypic antiretroviral drug resistance testing at low viral loads in the UK. HIV Med. 9, 673–676. doi: 10.1111/j.1468-1293.2008.00607.x, PMID: 18557948

[ref7] Chowdhury M. T. Bershteyn A. Milali M. Citron D. Nyimbili S. Musuka G. (2023). Progress towards UNAIDS's 95-95-95 targets in Zimbabwe: sociodemographic constraints and geospatial heterogeneity. medRxiv 202310.1101/2023.07.26.23293207

[ref8] de Felipe B. Pérez-Romero P. Abad-Fernández M. Fernandez-Cuenca F. Martinez-Fernandez F. J. Trastoy M.. (2011). Prevalence and resistance mutations of non-B HIV-1 subtypes among immigrants in southern Spain along the decade 2000–2010. Virol. J. 8, 1–7. doi: 10.1186/1743-422X-8-416, PMID: 21871090 PMC3170306

[ref9] Escudero D. J. Marukutira T. McCormick A. Makhema J. Seage G. R. III (2019). Botswana should consider expansion of free antiretroviral therapy to immigrants. J. Int. AIDS Soc. 22:e25328. doi: 10.1002/jia2.25328, PMID: 31190456 PMC6562114

[ref10] Faturiyele I. Karletsos D. Ntene-Sealiete K. Musekiwa A. Khabo M. Mariti M. (2018). Access to HIV care and treatment for migrants between Lesotho and South Africa: a mixed methods study. BMC Public Health 18:668. doi: 10.1186/s12889-018-5594-3, PMID: 29843667 PMC5975397

[ref11] Fennell C. Escudero D. Zash R. Diseko M. Mayondi G. Mabuta J.. (2023). The impact of free antiretroviral therapy for pregnant non-citizens and their infants in Botswana. J. Int. AIDS Soc. 26:e26161. doi: 10.1002/jia2.26161, PMID: 37885157 PMC10603275

[ref12] Gaolathe T. Wirth K. E. Holme M. P. Makhema J. Moyo S. Chakalisa U.. (2016). Botswana’s progress toward achieving the 2020 UNAIDS 90-90-90 antiretroviral therapy and virological suppression goals: a population-based survey. Lancet HIV 3, e221–e230. doi: 10.1016/S2352-3018(16)00037-0, PMID: 27126489 PMC5146754

[ref13] Gonzalez-Serna A. Min J. E. Woods C. Chan D. Lima V. D. Montaner J. S. G.. (2014). Performance of HIV-1 drug resistance testing at low-level viremia and its ability to predict future virologic outcomes and viral evolution in treatment-naive individuals. Clin. Infect. Dis. 58, 1165–1173. doi: 10.1093/cid/ciu019, PMID: 24429436 PMC3967823

[ref14] Government of Botswana: Botswana AIDS impact survey V 2021 (BAIS V): report., in national AIDS & health promotion agency, (2023), Gaborone, Botswana

[ref15] Grabowski M. K. Lessler J. Redd A. D. Kagaayi J. Laeyendecker O. Ndyanabo A.. (2014). The role of viral introductions in sustaining community-based HIV epidemics in rural Uganda: evidence from spatial clustering, phylogenetics, and egocentric transmission models. PLoS Med. 11:e1001610. doi: 10.1371/journal.pmed.1001610, PMID: 24595023 PMC3942316

[ref16] Grabowski M. K. Redd A. D. (2014). Molecular tools for studying HIV transmission in sexual networks. Curr. Opin. HIV AIDS 9:126. doi: 10.1097/2FCOH.0000000000000040, PMID: 24384502 PMC4109889

[ref17] HIV DRUG RESISTANCE REPORT 2021. (2021), World health organization Geneva

[ref18] IN DANGER: UNAIDS Global AIDS Update 2022 (2022). Geneva:Joint United Nations Programme on HIV/AIDS (UNAIDS), 2022.

[ref19] Ji H. (2022). Current research on HIV drug resistance—a topical collection with “pathogens”. Pathogens 11:966. doi: 10.3390/pathogens11090966 36145398 PMC9504728

[ref20] Kate Grabowski M. Lessler J. Bazaale J. Nabukalu D. Nankinga J. Nantume B.. (2020). Migration, hotspots, and dispersal of HIV infection in Rakai, Uganda. Nat. Commun. 11:976. doi: 10.1038/s41467-020-14636-y, PMID: 32080169 PMC7033206

[ref21] Kelentse N. Moyo S. Choga W. T. Lechiile K. Leeme T. B. Lawrence D. S.. (2023). High concordance in plasma and CSF HIV-1 drug resistance mutations despite high cases of CSF viral escape in individuals with HIV-associated cryptococcal meningitis in Botswana. J. Antimicrob. Chemother. 78, 180–184. doi: 10.1093/jac/dkac372, PMID: 36322466 PMC10205474

[ref22] Kouamou V. Washaya T. Ndhlovu C. E. Manasa J. (2023). Low prevalence of pre-treatment and acquired drug resistance to Dolutegravir among treatment naive individuals initiating on Tenofovir, lamivudine and Dolutegravir in Zimbabwe. Viruses 15:91882. doi: 10.3390/v15091882 PMC10534864 37766288

[ref23] Lurie M. N. Williams B. G. (2014). Migration and health in southern Africa: 100 years and still circulating. Health Psychol. Behav. Med. Open Access J. 2, 34–40. doi: 10.1080/21642850.2013.866898, PMID: 24653964 PMC3956074

[ref24] Maggiorella M. T. S. N. Brindicci G. Monno L. Santoro C. R. Coppola N. Cuomo N.. (2020). High HIV-1 diversity in immigrants resident in Italy (2008–2017). Sci. Rep. 10:3226. doi: 10.1038/s41598-020-59084-2 32094387 PMC7039940

[ref25] Maruapula D. Seatla K. K. Morerinyane O. Molebatsi K. Giandhari J. de Oliveira T.. (2022). Low-frequency HIV-1 drug resistance mutations in antiretroviral naive individuals in Botswana. Medicine 101:e29577. doi: 10.1097/MD.0000000000029577, PMID: 35838991 PMC11132386

[ref26] Marukutira T. Block L. Alwano M. G. Behel S. Jarvis J. N. Chakalisa U.. (2019a). Comparison of knowledge of HIV status and treatment coverage between non-citizens and citizens: Botswana combination prevention project (BCPP). PLoS One 14:e0221629. doi: 10.1371/journal.pone.0221629, PMID: 31465494 PMC6715216

[ref27] Marukutira T. Scott N. Kelly S. L. Birungi C. Makhema J. M. Crowe S.. (2020). Modelling the impact of migrants on the success of the HIV care and treatment program in Botswana. PLoS One 15:e0226422. doi: 10.1371/journal.pone.0226422, PMID: 31940360 PMC6961860

[ref28] Marukutira T. Stoové M. Lockman S. Mills L. A. Gaolathe T. Lebelonyane R.. (2018). A tale of two countries: progress towards UNAIDS 90-90-90 targets in Botswana and Australia. J. Int. AIDS Soc. 21:e25090. doi: 10.1002/jia2.25090 29508945 PMC5838412

[ref29] Marukutira T. Yin D. Cressman L. Kariuki R. Malone B. Spelman T.. (2019b). Clinical outcomes of a cohort of migrants and citizens living with human immunodeficiency virus in Botswana: implications for joint united nation program on HIV and AIDS 90-90-90 targets. Medicine 98:e15994. doi: 10.1097/2FMD.0000000000015994, PMID: 31169739 PMC6571245

[ref30] Marukutira T. Alwano M. G. Behel S. Jarvis J. N. Chakalisa U. Powis K. (2017). Immigrants and Botswana's ART program: potential barriers to epidemic control. Presented at the conference on retroviruses and opportunistic infections, Seattle, February 13–16, (2017). Abstract 2017

[ref31] McAuliffe M. Triandafyllidou A. International Organization for Migration (IOM) (2021). Migration research and analysis: recent United Nations contributions. In: world migration report 2022. World Migration Report, 2022 2022:e00025.

[ref32] Migrant-refugees: Botswana migration profile, in migrants & refugees section. (2023), Available at: www.migrants-refugees.va

[ref33] Mine M. Stafford K. Laws R. L. Marima R. Lekone P. Ramaabya D. (2021). Botswana Achieved the joint united nations programme on HIV/AIDS (UNAIDS) 95-95-95 targets: results from the fifth Botswana HIV/AIDS impact survey (BAIS V). In the 24th International AIDS Conference. Montreal, Canada

[ref34] Moyo S. Gaseitsiwe S. Zahralban-Steele M. Maruapula D. Nkhisang T. Mokaleng B.. (2019). Low rates of nucleoside reverse transcriptase inhibitor and nonnucleoside reverse transcriptase inhibitor drug resistance in Botswana. AIDS 33:1073. doi: 10.1097/2FQAD.0000000000002166, PMID: 30946161 PMC6467559

[ref35] Novitsky V. Bussmann H. Logan A. Moyo S. van Widenfelt E. Okui L.. (2013). Phylogenetic relatedness of circulating HIV-1C variants in Mochudi, Botswana. PLoS One 8:e80589. doi: 10.1371/journal.pone.0080589, PMID: 24349005 PMC3859477

[ref36] Pimentel V. Pingarilho M. Alves D. Diogo I. Fernandes S. Miranda M.. (2020). Molecular epidemiology of HIV-1 infected migrants followed up in Portugal: trends between 2001–2017. Viruses 12:268. doi: 10.3390/v12030268, PMID: 32121161 PMC7150888

[ref37] Richman D. D. (2014). Editorial commentary: extending HIV drug resistance testing to low levels of plasma viremia. Clin. Infect. Dis. 58, 1174–1175. doi: 10.1093/cid/ciu025, PMID: 24429434

[ref38] Rowley C. F. MacLeod I. J. Maruapula D. Lekoko B. Gaseitsiwe S. Mine M. (2016). Sharp increase in rates of HIV transmitted drug resistance at antenatal clinics in Botswana demonstrates the need for routine surveillance. J. Antimicrob. Chemother. 71, 1361–1366. doi: 10.1093/jac/dkv500, PMID: 26929269 PMC4830419

[ref39] Schultz C., World migration report. Migartion, health, and cities migration, health and urbanization: interrelated challenges Berlin. Berlin: International Organization for Migration (IOM), (2014), Available at: https://www.who.int/teams/health-and-migration-programme/world-report-on-the-health-of-refugees-and-migrants

[ref40] Seatla K. K. Choga W. T. Mogwele M. Diphoko T. Maruapula D. Mupfumi L.. (2019). Comparison of an in-house ‘home-brew’and commercial ViroSeq integrase genotyping assays on HIV-1 subtype C samples. PLoS One 14:e0224292. doi: 10.1371/journal.pone.0224292, PMID: 31751353 PMC6871785

[ref41] Shafer R. W. Frenkel L. M. (2019). The clinical implications of pretreatment drug resistance—a moving target, vol. 69 Oxford University Press US, 215–217. 10.1093/cid/ciy895 30321316

[ref42] Song J. Okano J. T. Ponce J. Busang L. Seipone K. Valdano E. (2023). The role of migration networks in the development of Botswana’s generalized HIV epidemic. elife 12:e85435. doi: 10.7554/eLife.85435 37665629 PMC10476964

[ref43] Stover J. Glaubius R. Teng Y. Kelly S. Brown T. Hallett T. B.. (2021). Modeling the epidemiological impact of the UNAIDS 2025 targets to end AIDS as a public health threat by 2030. PLoS Med. 18:e1003831. doi: 10.1371/journal.pmed.1003831, PMID: 34662333 PMC8559943

[ref44] Summary Sheet, Zimbabwe population-based HIV impact assessment [internet]. PHIA Project. U.S. Embassy in Zimbabwe. (2020), Available at: https://phia.icap.columbia.edu/countries/zimbabwe/

[ref45] Tanser F. Bärnighausen T. Vandormael A. Dobra A. (2015). HIV treatment cascade in migrants and mobile populations. Curr. Opin. HIV AIDS 10, 430–438. doi: 10.1097/COH.0000000000000192 26352396

[ref46] The path that ends AIDS: UNAIDS Global AIDS Update 2023. Geneva: Joint United Nations Programme on HIV/AIDS. (2023), Available at: https://www.unaids.org/en/resources/documents/2023/global-aids-update-2023

[ref47] Vinie Kouamou A. M. M. (2021). High levels of pre-treatment HIV drug resistance in Zimbabwe: is this a threat to HIV/AIDS control? J. AIDS HIV Treatment. 3, 42–45. doi: 10.33696/AIDS.3.021

[ref48] Wise J. (2023). Ending AIDS by 2030 is a political choice, says UN agency. BMJ 382:p1667. doi: 10.1136/bmj.p1667 37468149

[ref49] Wymant C. Bezemer D. Blanquart F. Ferretti L. Gall A. Hall M.. (2022). A highly virulent variant of HIV-1 circulating in the Netherlands. Science 375, 540–545. doi: 10.1126/science.abk1688 35113714

[ref50] Yebra G. de Mulder M. Holguín Á. (2013). Description of HIV-1 group M molecular epidemiology and drug resistance prevalence in Equatorial Guinea from migrants in Spain. PLoS One 8:e64293. doi: 10.1371/journal.pone.0064293, PMID: 23717585 PMC3661467

[ref51] Zhao B. Song W. Kang M. Dong X. Li X. Wang L.. (2022). Molecular network analysis reveals transmission of HIV-1 drug-resistant strains among newly diagnosed HIV-1 infections in a moderately HIV endemic city in China. Front. Microbiol. 12:797771. doi: 10.3389/fmicb.2021.797771, PMID: 35069498 PMC8778802

